# Self-care in the person with chronic disease: A protocol for a self-care intervention

**DOI:** 10.1016/j.mex.2024.102829

**Published:** 2024-07-02

**Authors:** Maria Marques, Catarina Martins, Margarida Goes, Ana Escoval, Vanessa Nicolau, Lara Pinho, Henrique Oliveira, Isabel Bico, Susana Mendonça, José Moreira, Rute Pires, Miguel Pedrosa, Cláudia Mendes, César Fonseca

**Affiliations:** a CHRC - Comprehensive Health Research Centre, Universidade de Évora, Évora, Portugal; b Escola Superior de Enfermagem São João de Deus, Universidade de Évora, Évora, Portugal; c Universidade de Évora, Évora, Portugal; d Instituto de Telecomunicações, Aveiro, Portugal; e Escola Nacional de Saúde Pública, Lisboa, Portugal; f Hospital Espírito Santo de Évora - Unidade Local de Saúde do Alentejo Central, Évora, Portugal; g CBIOS – Universidade Lusófona's Research Center for Biosciences & Health Technologies Lisbon*,* Portugal

**Keywords:** Cláudia Mendes, Self-care, Chronic disease, Chronic illness, Patient, Nursing

## Abstract

Most aging populations report chronic illnesses, which are usually permanent or recurrent, significantly affect well-being and quality of life, require daily and consistent healthcare management, and last more than three months. Improved health outcomes and reduced healthcare costs are associated with self-care in treating chronic illnesses. The aim is to describe a protocol using a self-care intervention in a person with a chronic disease. A longitudinal study will be conducted with 40 patients. This article describes a protocol for a self-care intervention in a person with a chronic disease. The outcome measures will be compared with measures after the intervention in three different chronologic times. Randomization will be used to assign participants to the intervention group. The present study is expected to generate significant information about the role of self-care intervention in persons with chronic disease.

## Graphical abstract

**Figure:**
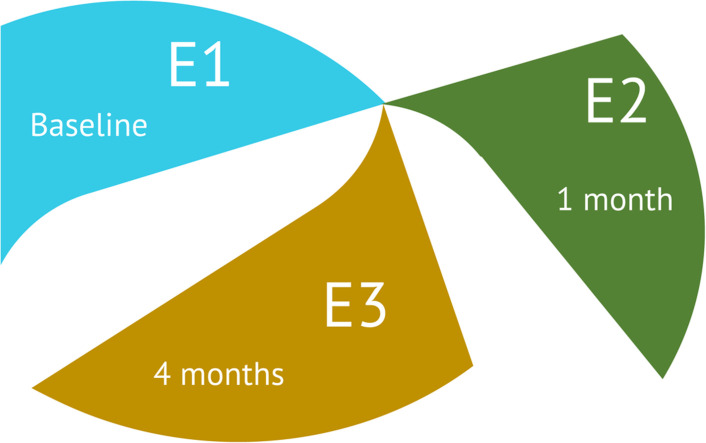

Specifications table

**Table:** 

Subject area	Medicine and Dentistry
More specific subject area	Lifestyles and health care; Health policies and research in health services; Research in patient-centered care
Name of your protocol	SELF-CARE IN THE PERSON WITH CHRONIC DISEASE
Reagents/tools	SELF-CARE OF CHRONIC ILLNESS INVENTORY WHOQOL-BREF(PT) CORE SET
Experimental design	A quantitative longitudinal study with 400 patients randomly selected. A protocol using a self-care intervention with adults registered in consultations of diabetes, pulmonology, and cardiology who have more than one chronic disease in the Hospital. In two different chronologic times, baseline measures will be compared with measures after self-care intervention. Participants' randomization will be determined to assign them to the intervention group.
Trial registration	The study protocol was registered in the Clinicaltrials.gov NCT06235593
Ethics	The study protocol was submitted to the Research Ethics Committee of the University and Hospital and approved under No. 20/23
Value of the protocol	• This protocol is essential to determine the nursing care needs of the elderly population sample according to the self-care deficits identified in the elderly population living in their own homes or the homes of relatives or friends in the district of Évora. • There needs to be more knowledge about the effects of self-care interventions. • This article is essential to contribute to the recommendations for self-care for older adults.

## Description of protocol

Chronic diseases affect at least 59 % of the Portuguese population. About 18 % of users of the National Health System have one chronic disease, 11 % have two, 8 % have three, and 22 % have four or more chronic diseases. Multimorbidity increases with age, so a continuous and accessible care model is essential for elderly individuals. Self-care behavior is crucial for improved health-related quality of life, and the self-care process involves maintenance, monitoring, and management [1]. The ``Self-Care of Chronic Illness Inventory—Patient Version'' is a generic measure designed to assess the self-care process. Overall, managing chronic conditions through self-care is critical in reducing healthcare costs. [2].

According to Barbara Riegel et al., self-care is essential in aging, mainly when changes occur in the person's health condition. Theoretically, this process involves three linked sequential behaviors captured in the critical concepts of self-care maintenance, self-care monitoring, and self-care management. Self-care maintenance addresses behaviors patients with a chronic illness use to maintain physical and emotional stability (e.g., medication adherence). In contrast, self-care monitoring involves observing oneself for signs and symptoms (e.g., checking blood pressure): self-care monitoring links or bridges between self-care maintenance and self-care management. A core goal of self-care monitoring is symptom recognition; once recognized, self-care management (e.g., taking medicine for a symptom) can occur, with behaviors that reflect a response to the symptoms observed. There are two approaches to measure self-care: generic and disease specific. Generic measures are applicable to a wide range of patients, while disease-specific measures are useful in specific groups with a single condition. Disease-specific measures are more responsive because they target issues experienced by patients with a specific condition. In contrast, generic measures allow comparison among patients with different conditions and accommodate those with more than one diagnosis [3].

## Objectives

### Primary objectives

- To analyze the effects of a self-care intervention managed by nurses on the self-management of chronic diseases.

Secondary objectives

- To characterize how a specialized intervention can contribute to changes in lifestyles, increased well-being and self-care of patients with chronic diseases.
- To promote self-care maintenance, monitoring and management.

### Study design

We have designed a longitudinal and prospective study to evaluate an intervention's effects on patients with chronic disease in a Hospital on Portugal. During the medical consultation, the patients will be invited to participate in the study and will receive a free and detailed consent form to sign if they agree to participate. We have followed the SPIRIT 2013 recommendations to ensure that our study protocol is rigorous and comprehensive.

### Subjects

Participants will be selected from the patients registered in consultations of diabetes, pulmonology, and cardiology who have more than one chronic disease in the Hospital.

### Eligibility criteria

The inclusion criteria were: individuals who (i) are aged 65 or older; (ii) are interested in participating in the project; (iii) are residing in the Évora district in their own homes or at family members' or friends' homes; (iv) patients who are sick or are hospitalized due to acute, short-term health needs, make their own decisions; and (v) present at least one chronic disease.

### Sample size calculation

Considering the explorative nature of the study, a formal sample size analysis is not possible. However, we expected to collect 40 patients, selected from the hospital database based on the G*Power software. The number of participants was calculated as 33 cases by using linear multiple regression with *α* = 0.05 and 1 − *β* = 0.80. Considering a dropout rate of 20 %, the minimum sample size was therefore derived as 40 patients.

### Recruitment

The invitation to participate will be made in the context of consultation, and subjects who agree to participate in the study will be given the free and informed consent form previously approved by the University and Hospital Ethics Committee.

### Outcomes and variables

Immediately after recruitment, all enrolled patients who agree to participate will sign the informed consent.

**Fig. 1:**
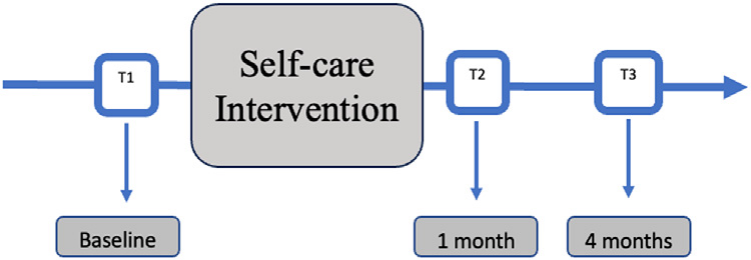
Intervention schedule.

The subjects then proceed to the baseline data collection, intervention protocol, and final assessments (Fig. 1).

In the first contact, we evaluated functionality, health-related quality of life, and Self-Care of Chronic Illness Inventory—Patient Version, with three linked sequential behaviors captured in the critical concepts of self-care maintenance, monitoring, and management. Self-care maintenance addresses behaviors patients with a chronic illness use to maintain physical and emotional stability (e.g., medication adherence). In contrast, self-care monitoring involves observing oneself for signs and symptoms (e.g., checking blood pressure): self-care monitoring links or bridges between self-care maintenance and self-care management.

We will conduct a mixed methods evaluation, including a quantitative outcome evaluation to assess the effectiveness of the intervention and a qualitative and quantitative process evaluation to derive indications for future implementation in different clinical settings (Table 1).

- Anthropometry: Weight evaluation will be done using a scale and height of a stadiometer. The International Society for the Advancement of Kinanthropometry (ISAK) protocol assessed waist circumference.
- Chronic diseases will be evaluated based on the number, medication and measure evaluation.
- ``Self-Care of Chronic Illness Inventory - Patient Version'' (version 4) instrument which includes 20 items across three scales: (i) Self-Care Maintenance (8 items); (ii) Self-Care Monitoring (6 items); and (ii) Self-Care Management (6 items). All the responses are based on a 5-point Likert scale, with one being ``Never'' or ``Not Likely'' and five being ``Always'' or ``Very Likely'' [2];
- The ``WHOQOL-BREF(PT)'' instrument, which comprises 24 facets (one item per facet) clustered into four domains, allows for the measurement of the quality of life of people following the World Health Organization criteria [4].
- The ``CORE SET'' (version 4) instrument, the Elderly Nursing Core Set'', aims to classify the degree of elderly functioning, which is also capable of even establishing elderly ``nursing care needs'' [5,6].

**Table 1:** Measures and outcomes.

Measures and outcomes	Tools of assessment	Data source
Sociodemographic data	• Sociodemographic profile	- Sex, age, educational level
Anthropometric	• Weight scale • Stadiometer • Abdominal measuring tape • Body mass index	- Weight - Height - Abdominal circumference
Chronic diseases	• Medication • Sphygmomanometer • Blood samples • Glycemia dispositive • Finger oxygen device	- Number of diseases - Chronic medication - Mean blood pressure - Glycemia variation - Blood oxygen level
Health-related quality of life	• IWQOL-BREF	- 24 facets (one item per facet) clustered into four domains, allows for the measurement of the quality of life of people following the World Health Organization criteria
Self-care	• Self-Care Inventory of Chronic Illness Inventory – Patient Version	- 20 items across three scales: (i) Self-Care Maintenance (8 items); (ii) Self-Care Monitoring (6 items); and (ii) Self-Care Management (6 items). All the responses are based on a 5-point Likert scale, with one being ``Never'' or ``Not Likely'' and five being ``Always'' or ``Very Likely''
Elderly nursing core set (ENCS)	• Elderly functioning	- 28-item, self-report questionnaire that aims to investigate attitudes, behaviors and cognitions related to eating disorder symptoms

### Intervention description

A disease management and patient empowerment program will be developed based on quality standards. This program will promote self-care by encouraging specialist nurses to adopt an anticipatory approach and follow professional responsibility and prudence principles. Monitoring and intervention will focus on recognizing and empowering others by building interpersonal relationships and helping them develop knowledge and self-confidence. A specialist nurse will provide patients in the intervention group with management and lifestyle information. The self-care intervention program will include a range of techniques for managing different chronic diseases based on reports from older individuals with chronic diseases. The program consists of two sessions daily, with at least one month between each (Table 2, Table 3).

**Table 2:** Intervention planning.

	Unit of competence	Concepts of self-care	Self-care behaviors
Session 1	• Empowers the person and family/significant other, for the management of treatment • Promotes care for the person with chronic disease	Self-care maintenance Self-care monitoring Self-care management	- Lifestyle improvement - Motivation - Treatment adherence - Medication management - Comorbidities management - Physical activity - Hydration - Weight control - Body composition management - Smoking cessation - Alcohol use - Sleep improvement - Dietary intake - Psychosocial consequences
Session 2	• Empowers the person and family/significant other, for the management of treatment • Promotes care for the person with chronic disease	Self-care monitoring Self-care management	- Activity changes - Weight control - Body composition management - Managing symptoms - Medication changes - Smoking cessation - Alcohol use - Psychosocial consequences - Pain management - Managing dietary changes - Consulting healthcare provider - Nausea and vomiting management

**Table 3:** Self-care protocol.

1st evaluation	Baseline

2nd Evaluation	One month (post-intervention)
3rd Evaluation	Four months (post-intervention)

## Evaluation

We have three evaluations: baseline (before intervention), one and four months after intervention.

## Discussion

This is an innovative work that will allow this Research Center to be at the forefront of a pioneering work that can significantly contribute to the promotion of scientific research in the field of active and healthy aging research, improving the survey of needs, the development, monitoring, and Evaluation of nursing interventions, and the dissemination of good practices and innovation in older people with chronic diseases (multimorbidity) [7].

This project meets a new paradigm that has been assuming more significant and critical relevance: the construction of a ``health value model'' that aims to obtain results that promote positive changes in people's health conditions [8,9]. The form of Evaluation of this model is based on the recording of data throughout the care cycle. It focuses primarily on people's expectations and how much care has contributed (or not) to improving their health status.

The concept of self-care underlies nursing interventions at different levels and allows for the organization of care according to the needs of people aged 65 and older presenting multimorbidity [10]. Older people need personalized and comprehensive care, given the specificity of the health condition of the older person and, indirectly, through education regarding self-care, i.e., the promotion of good practices. Its scope may focus on health promotion or disease prevention, together with the older person and family caregiver, in managing their health condition, preferably at home (home-based care).

## Statistical methods

Several statistical analysis techniques will be used: psychometric, descriptive, and inferential. Confirmatory Factor Analysis techniques (application of Structural Equation Models), Multiple Linear Regression Methods and Ordinal Regression Methods will also be used. A confidence level of 95 % and an error of 4.5 % will be considered.

## Ethics statements

The work described has been carried out by the Ethical Committee of the University and Ethical Professional of the Hospital, approved with ethics protocol number 20/23. Participation was voluntary, with written informed consent obtained from all participants.

## CRediT authorship contribution statement

**Maria Marques:** Conceptualization, Methodology, Software, Data curation, Writing – original draft, Supervision. **Catarina Martins:** Visualization, Investigation. **Margarida Goes:** Supervision, Software, Validation. **Ana Escoval:** Supervision, Software, Validation. **Vanessa Nicolau:** Supervision, Software, Validation. **Lara Pinho:** Visualization, Investigation. **Henrique Oliveira:** Supervision, Software, Validation. **Isabel Bico:** Visualization, Investigation. **Susana Mendonça:** Visualization, Investigation. **José Moreira:** Visualization, Investigation. **Rute Pires:** Supervision, Software, Validation. **Miguel Pedrosa:** Visualization, Investigation. **Cláudia Mendes:** Conceptualization, Methodology, Software, Data curation, Writing – original draft. **César Fonseca:** Supervision, Software, Validation.

## Declaration of competing interest

The authors declare that they have no conflict of interest.

## Data Availability

Data will be made available on request.
